# Supplementary material for: Coherent Vibrational Wave Packet Motion in ErCry4a Proteins Monitors the Redox State of the Flavin Chromophore
Source: J Am Chem Soc. 2026 Sep 24;148(39):42632–44. doi: 10.1021/jacs.6c14527 (PMC13651507; doi:10.1021/jacs.6c14527)
Supplement: Supplementary file 1 [file ja6c14527_si_001.pdf]

Supporting Information for

Coherent vibrational wave packet motion in *Er*Cry4a proteins monitors the redox state of the flavin chromophore

*Krishan Kumar,[a] Daniel Timmer,[a] Maryam Khazani,[b] Glen Dautaj,[b] Sophie Brinkmann,[b] Jan P. Götze,[c] Peter Saalfrank,[d] Boris F. Gribakin,[a] Antonietta De Sio,[a,e] Henrik Mouritsen,[b,f] and Christoph Lienau[a,e,f]**

[a] Institut für Physik, Carl von Ossietzky Universität Oldenburg, 26129 Oldenburg, Germany

[b] AG Neurosensory Sciences/Animal Navigation, Institut für Biologie und Umweltwissenschaften, Carl von Ossietzky Universität Oldenburg, 26129 Oldenburg, Germany

[c] Institut für Chemie und Biochemie, Freie Universität Berlin, 14195 Berlin, Germany

[d] Institute of Chemistry, University of Potsdam, 14476 Potsdam, Germany

[e] Center for Nanoscale Dynamics (CENAD), Carl von Ossietzky Universität Oldenburg, 26129 Oldenburg, Germany

[f] Research Center for Neurosensory Sciences, Carl von Ossietzky Universität Oldenburg, 26111 Oldenburg, Germany

*Correspondence to: christoph.lienau@uni-oldenburg.de, henrik.mouritsen@uni-oldenburg.de

Content

1. Sample preparation 2
2. Experimental setup 2
3. Data evaluation 4
3.1. Time zero correction 4
3.2. Removal of XPM contributions 4
3.3. Global fit and Fourier analysis: 5
3.4. Vibrational frequencies deduced from pump-probe spectroscopy 9
3.5. Fourier maps 10
3.6. Theoretical calculations 13
4. References 16

1. Sample preparation

Wild-type *Er*Cry4a (GenBank: KX890129.1) was cloned, expressed, and purified according to the protocol described by Xu et al.[1] Briefly, the protein was expressed in BL21(DE3) *E. coli* cells and purified under red light conditions using Ni-NTA agarose columns, followed by anion-exchange chromatography. Purified protein samples were concentrated to 5 – 6 mg/mL, snap-frozen in liquid nitrogen, and lyophilized overnight. The resulting protein powder was stored at -80 °C until further use. Prior to the optical measurements, the protein samples were buffer exchanged into β-mercaptoethanol (BME)-free buffer (20 mM Tris-HCl, 200 mM NaCl, 20% (v/v) glycerol, pH 8.0) using a Millipore centrifugation filter (Amicon Ultra, 30 kDa) in a temperature-controlled microcentrifuge (4 °C, 13,500 rpm). The centrifugation steps also removes possible free FAD from the sample. To facilitate re-oxidation of the protein during the optical experiment, 2 mM potassium ferricyanide ($K_3Fe(CN)_6$, PFC) was added to the samples. Afterwards, we again centrifuged the protein for 1 h to remove possible protein aggregation. All pump–probe experiments on the protein were performed at a temperature of 4 °C and in the buffer solution at pH 8.0 to avoid photodegradation of the light-sensitive protein. The *Er*Cry4a mutants W_XF (X = A–D) were generated by replacing the DNA codon for tryptophan (TGG) at amino acid position 395 (W_AF), 372 (W_BF), 318 (W_CF), or 369 (W_DF) in the *Er*Cry4a gene by a phenylalanine codon (TTT) in a polymerase chain reaction using the Q5 site-directed mutagenesis kit (New England Biolabs), as described in detail in an earlier study.[2]

2. Experimental setup

Three different types of transient absorption setups were used. In the first set of measurements, 12-fs pump pulses centered around 450 nm are used together with optimally compressed probe pulses. In the second set, chirp-mirror compression was removed from the probe arm to avoid sinusoidal spectral phase modulations induced by multiple bounces off the chirped mirror. The experimental setup has been reported elsewhere.[3] In a third set of measurements, somewhat longer pump pulses were generated in an optical parametric amplifier and a white light supercontinuum extending into the UV was taken as a probe. This setup has been previously reported in Ref. 2.

All transient absorption measurements were performed using a regenerative amplifier (Legend Elite, Coherent) seeded by a Ti:sapphire oscillator (Vitara, Coherent), delivering 1 mJ pulses at 800 nm (26 fs, 10 kHz). The laser output was focused into a 1 m hollow-core fiber (Savana, Ultrafast Innovations) filled with 2.4 bar absolute pressure neon gas, generating a white-light supercontinuum (~350–1000 nm). The infrared portion was removed with a 700 nm short-pass filter (Edmund Optics). The output was split into pump and probe beams, and the pump was spectrally filtered using a 4-f grating filter and compressed with chirped mirrors (DCM12, Laser Quantum) and a wedge pair. The pump and the probe beams were temporally modulated using a chopper (MC2000B, Thorlabs) with a 2:1 duty cycle. The relative polarization and intensity of the pump beam were controlled using thin-film polarizers (Edmund Optics) and neutral density filters. The pump power and chopper state were monitored with a fast photodiode. The pump–probe delay was controlled by a motorized translation stage (M112.1DG1, Physik Instrumente). Both beams were focused into a 1 mm quartz cuvette (~40 µm spot size), and the transmitted probe was dispersed in a spectrograph (Acton SP-2150, Princeton Instruments) and detected with a high-speed line camera (Aviiva EM4, Teledyne e2v) at the full 10 kHz repetition rate. Differential transmission spectra $\Delta T/T$ were obtained from shot-to-shot measurements with scattering correction in the following form

$$\frac{\Delta T}{T}(t_d, \lambda) = \frac{S_{\text{on,on}} - S_{\text{off,on}} - S_{\text{on,off}}}{S_{\text{off,on}}}. \quad \text{(S1)}$$

In Eq. (S1), t_d denotes the waiting time between pump and probe and λ the wavelength of the probe laser. The spectrum recorded in the presence of pump and probe is denoted as $S_{\text{on,on}}$, while $S_{\text{on,off}}$ and $S_{\text{off,on}}$ are the spectra recorded with either the pump or the probe beam switched off. The pump spectrum was tuned to the spectral region ~435 – 500 nm using the above 4f spectral filter to ensure sufficient bandwidth for high time resolution. The chirped mirrors (DCM12, Laser Quantum) are unable to compress the spectral range below 435 nm; therefore, this spectral range was not used for photoexcitation.[4] Thus, the generated pump pulse was characterized using a home-built transient grating frequency-resolved optical gating[5] setup and is presented in an earlier report[3], yielding a retrieved pulse duration of ~12 fs. Additionally, to minimize the temporal duration of the cross-phase modulation (XPM) signals on the TA data around time zero, the probe beam was also compressed using another pair of chirped mirrors. The use of optimally compressed probe pulses largely facilitates the suppression of XPM signal around time zero, as discussed in Section 3.3. At the same time, however, multiple bounces on the chirp mirrors introduce higher-order, sinusoidal phase distortions on the pulse, as seen in the TG-FROG measurements in Fig. S1.

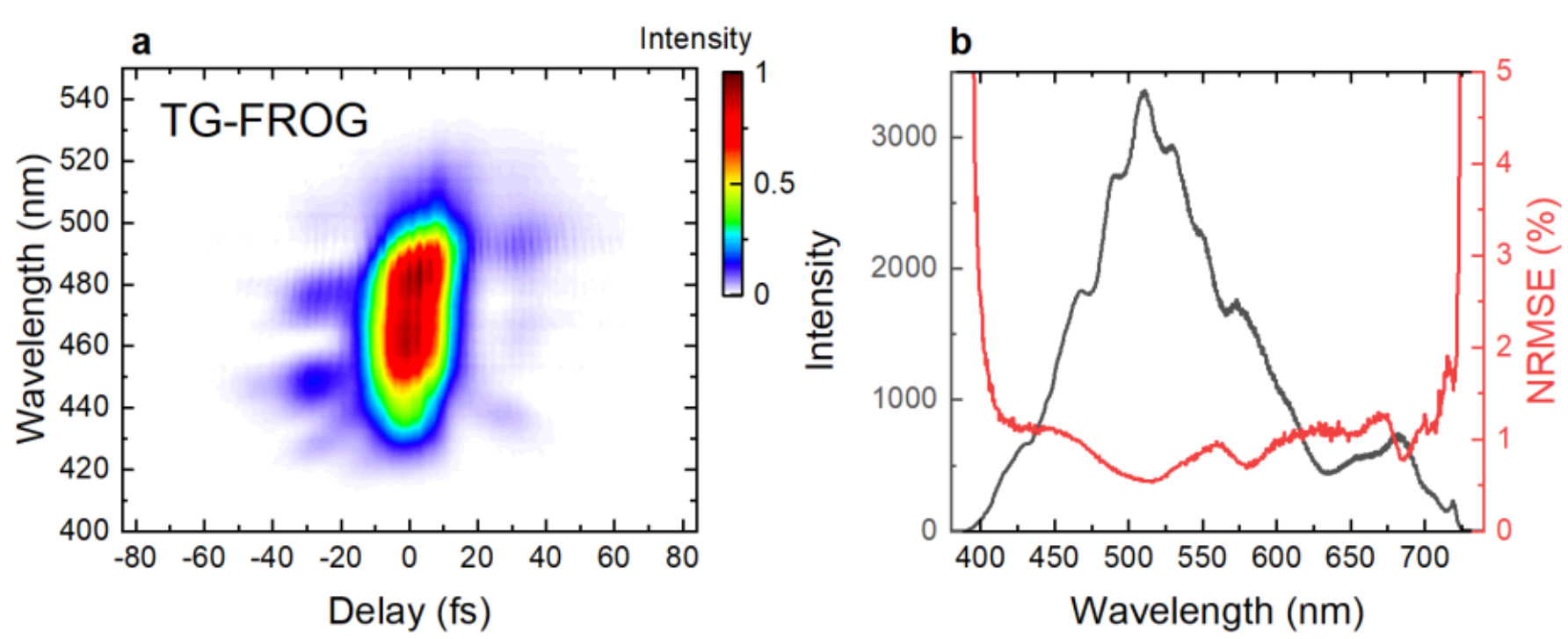

Figure S1*. Excitation pump pulse duration and laser stability. a) TG-FROG trace yielding a pulse duration of ~12 fs. b) Spectral single-shot stability measured for 1000 consecutive laser pulses using the Aviiva line camera, showing a normalized root mean square error of ~1%.*

The TA data reported in Figs. 1 and 2 of the main manuscript were recorded with such spectrally filtered 12-fs blue pump and optimally compressed probe pulses. This maximizes the time resolution of the experiment and allows us to gain insight into the nonadiabatic coupling dynamics within the first 50 fs. In addition it is the best configuration that we found to minimize the effects of cross-phase modulation[6] (XPM) on the ultrafast dynamics. The data were measured using with a relative polarization of pump and probe set to magic angle (54.7°), and with pump pulse energy of 25 nJ. Furthermore, such compressed probe measurements on protein and buffer were performed on the same day to facilitate quantitative comparison of the results. The TA datasets were recorded with a 5-fs step size, averaged over 1000 shots per time point, and extended to a pump-probe delay of ~3 ps. A series of such TA spectra was recorded and averaged by correcting for stage drift until a sufficient signal-to-noise ratio was obtained. The protein measurements were performed in the presence of PFC oxidizer. The control TA measurements therefore used a buffer solution containing 1 mM PFC. Under identical excitation conditions, PFC only shows a very weak nonlinear signal with a lifetime of a few picoseconds, and no detectable PL was observed.[2] The TA data were further processed as described in the data evaluation section.

In the analysis of the TA data, we found that a finite amount of sinusoidal chirp on the probe affects the Fourier spectra that are obtained from the coherent oscillations on these TA measurements. To avoid such complications and to facilitate a quantitative understanding of the coherently induced vibrational mode profiles, we additionally recorded TA data with chirped probe pulses. In these "chirped probe" measurements, we minimized possible higher-order phase contributions by removing the chirped mirrors from the probe arm. These measurements, reported in Fig. 3 of the main measurements, have been performed on a buffer solution that did not contain glycerol. This prevents a possible impulsive excitation of low-frequency modes of glycerol.

These "compressed" and "chirped" probe data are compared to measurements performed using the pump-probe setup described in Ref. [2]. Here, instead of using a white light supercontinuum, ~30 fs pump pulses centered at 450 nm are created in an optical parametric amplifier (TOPAS, Light Conversion). Probe pulses spanning the range from 320 nm to 750 nm are generated by focusing the fundamental of the regenerative amplifier onto a CaF_2 window that is constantly moved in lateral directions to avoid photodamage. The time resolution provided by the 30-fs pump pulses is sufficient for probing low-frequency coherent wavepacket motion but not for resolving the high-frequency modes. We used this setup because the extended probe spectral range offers more insights into the coupling of low-frequency vibrational modes to electronic transitions below 400 nm. More details about the experimental conditions and setup can be found in Ref. [2].

3. Data evaluation

3.1. Time zero correction

The chirped mirrors (DCM12, Laser Quantum) used in the present experiments do not compress the blue part above ~2.8 eV, therefore the remaining chirp is visible in the $\Delta T/T$ map recorded with compressed probe pulses. Since the XPM signal is stronger than the protein nonlinearity, it can be used both in the sample and reference measurement to determine the energy-dependent time zero. The probe-energy dependence of time zero that is deduced from these measurements is shown below as a black line in Fig. S2. This time zero measurement has been used to interpolate the different measurements onto the same delay time axis.

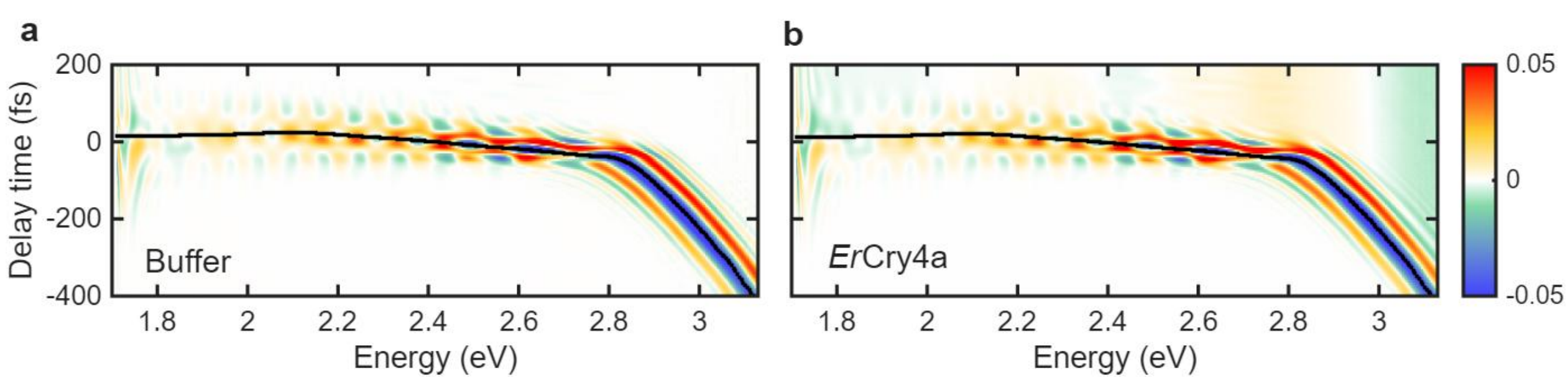

***Figure S2**. Time-zero correction of compressed differential transmission ($\Delta T/T$) maps. a) As measured pump-probe map of buffer measurement. b) Measured pump-probe map of protein in buffer. The deduced time-zero points are shown as black line on top of differential map.*

3.2. Removal of XPM contributions

As shown in Fig. S2, the reference measurement is performed on the buffer solution under identical experimental conditions as used in the protein measurement. We make use of this reference measurement to subtract strong XPM signals in the buffer to a good approximation from the protein

measurements. Fig. S3 shows the time corrected and XPM corrected $\Delta T/T$ signal at selected probe energies.

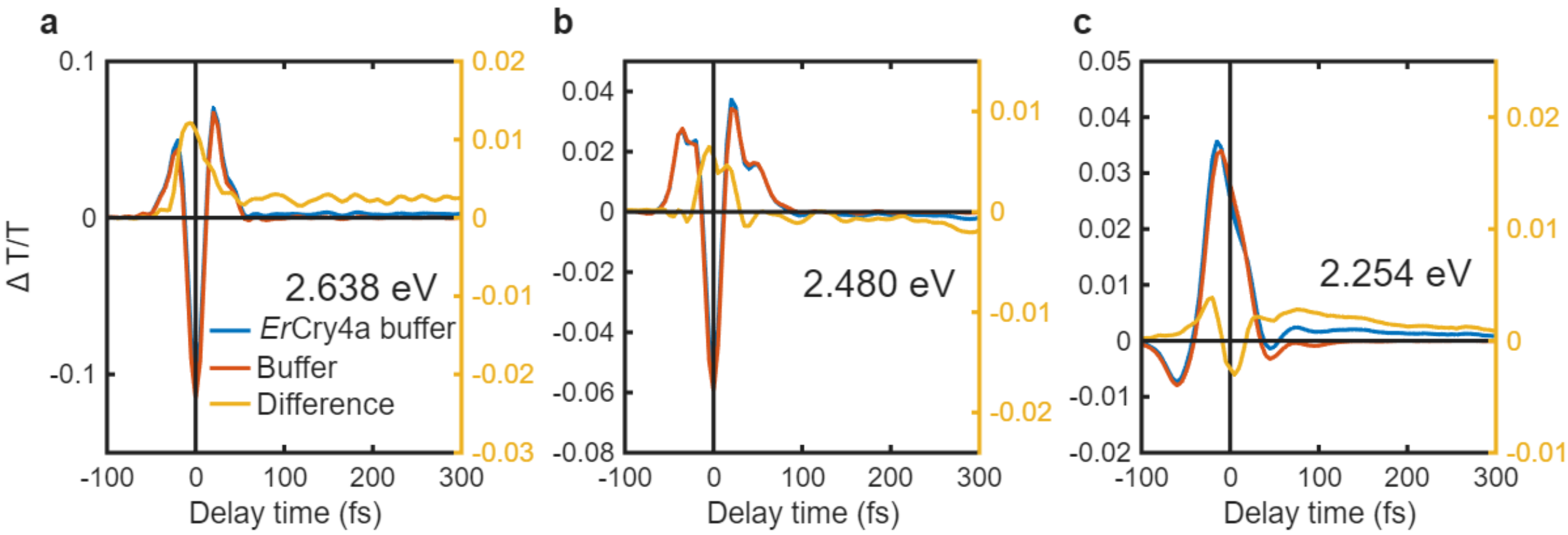

Figure S3. *Removal of XPM contribution for selected energies of a) 2.638 eV, b) 2.480 eV, and c) 2.254 eV. Reference $\Delta T/T$ measurements were performed under identical conditions on the buffer without protein. These data are shown, at selected probe energies, in orange. The corresponding protein data are plotted in blue on the same left y-axis. The buffer corrected $\Delta T/T$ signal is plotted on a different y-axis scale for clarity. The scale for the right y-axis is chosen such that the 0 matches on both left and right y-axis scales.*

The chirp corrected and solvent corrected differential map is shown in Fig. S4. The pump-probe data recorded with the chirped probe are corrected analogously.

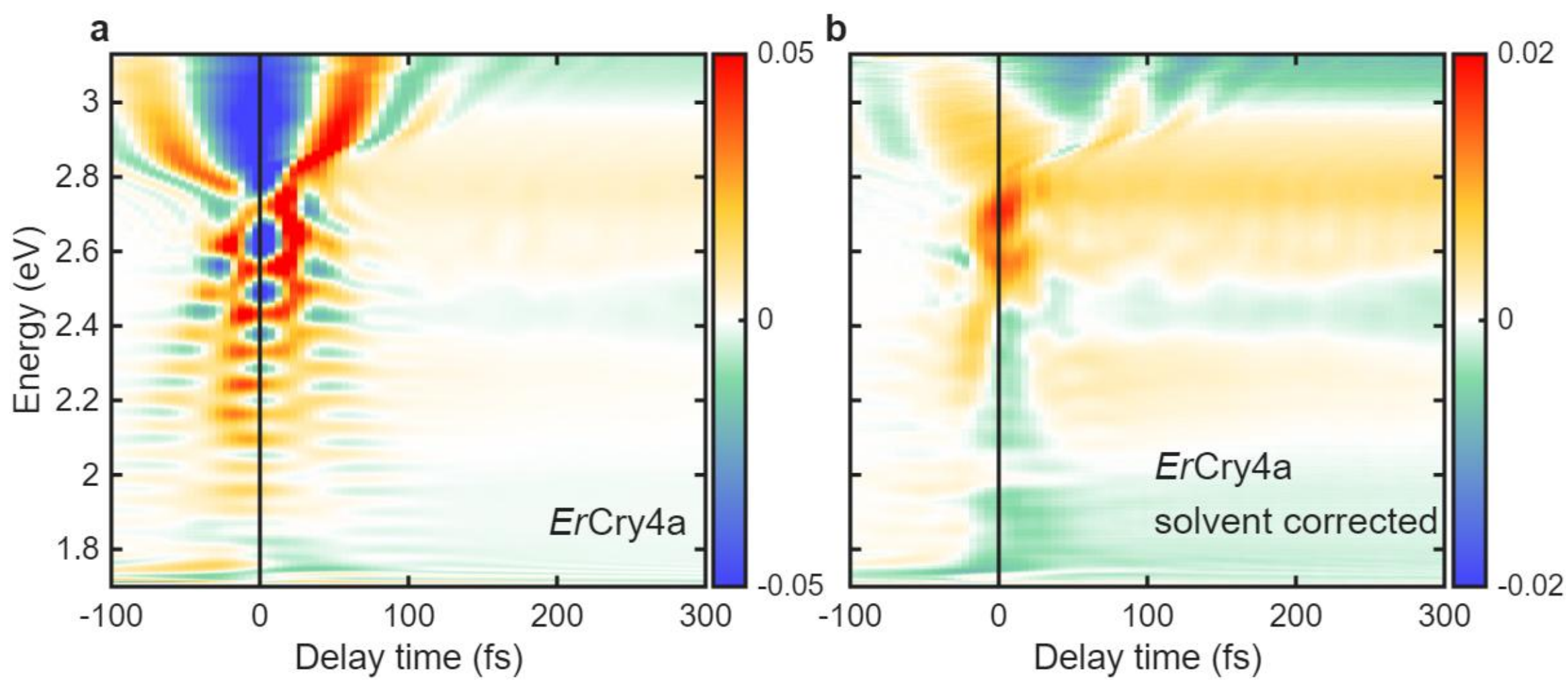

Figure S4. *$\Delta T/T$ map showing the Er*Cry4a *map before (a) and after (b) XPM subtraction.*

3.3. Global fit and Fourier analysis:

A global analysis is performed for the chirp and solvent corrected data recorded with compressed probe pulses in the delay range between 0 and 3000 fs range for probe energies between 1.7 – 3.1 eV. This analysis is done using a MATLAB-based toolbox as shown in the main text.[7] In the analysis, the $\Delta T/T$ measurements are approximated by three decay associated difference spectra (DADS) which decay exponentially in time with decay times of 16 fs, 360 fs and 80 ps, respectively.

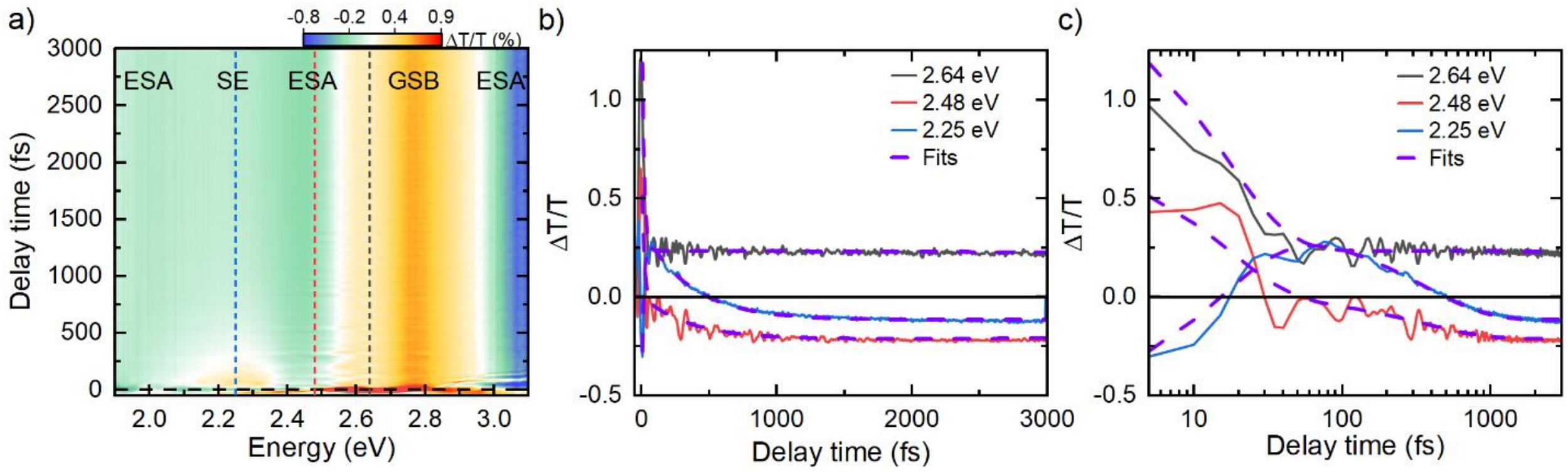

Figure S5*. Ultrafast spectroscopy of European Robin Cryptochrome (ErCry4a) protein dispersed in Tris buffer. (a) ΔT/T map of ErCry4a as a function of probe energy and delay time, shown for full recorded range of delay times. (b,c) ΔT/T spectra at selected delays plotted together with multiexponential fits obtained from global analysis. The data are plotted on a (b) linear and (c) logarithmic delay time axis.*

For analyzing coherent vibrational dynamics, we compute residual maps by subtracting the results of the global analysis from the experimental data.

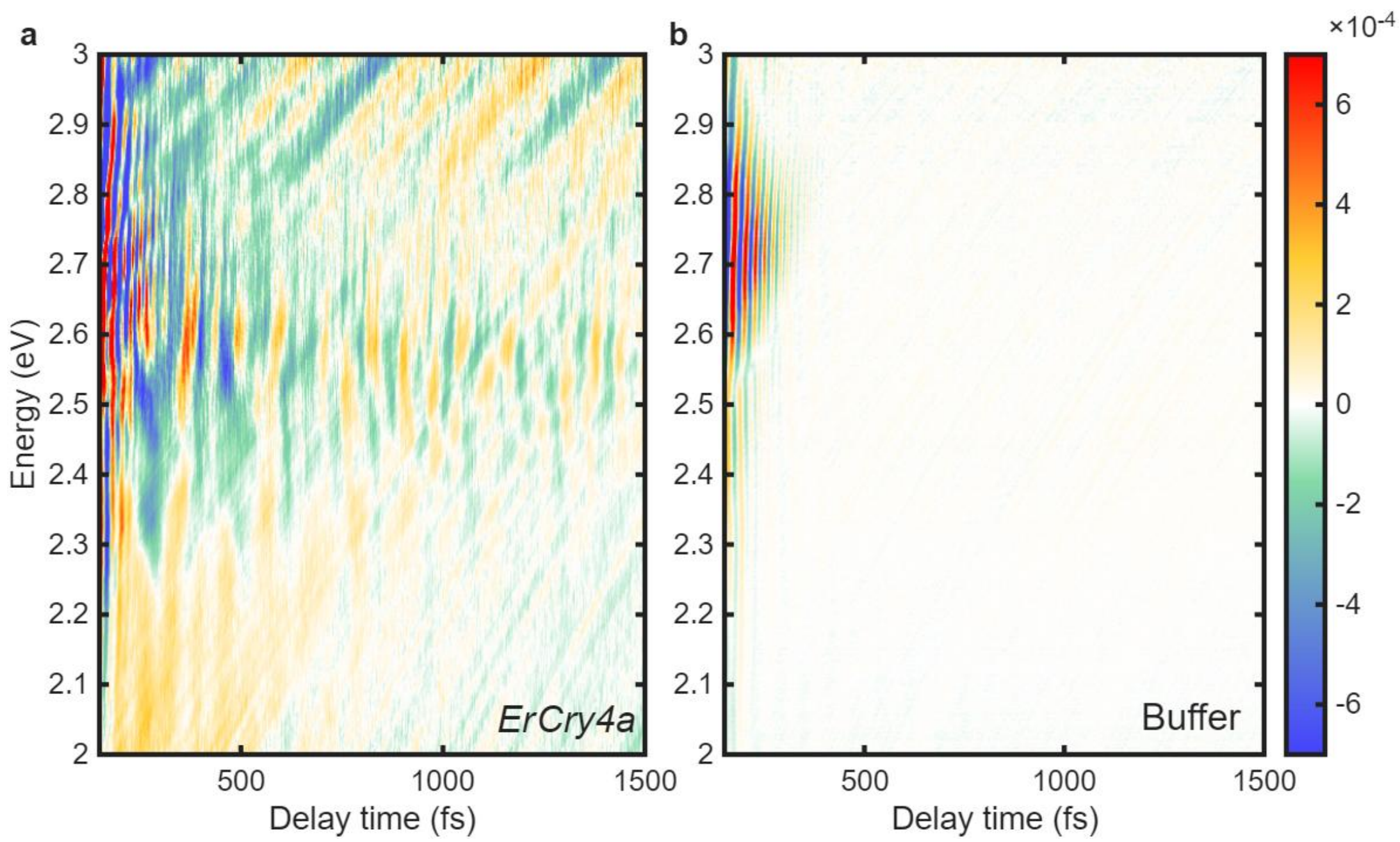

Figure S6*. Residual maps recorded using chirped probe pulses. The residual maps show the difference between the recorded $\Delta T/T$ and their fit to three exponentially decaying spectral components as a function of time delay and probe energy. The residual maps are shown for a)* ErCry4a *and b) from the reference measurement performed on the buffer solution. The buffer residuals are divided by 7 to display them on the same color scale as used in a) for better comparison.*

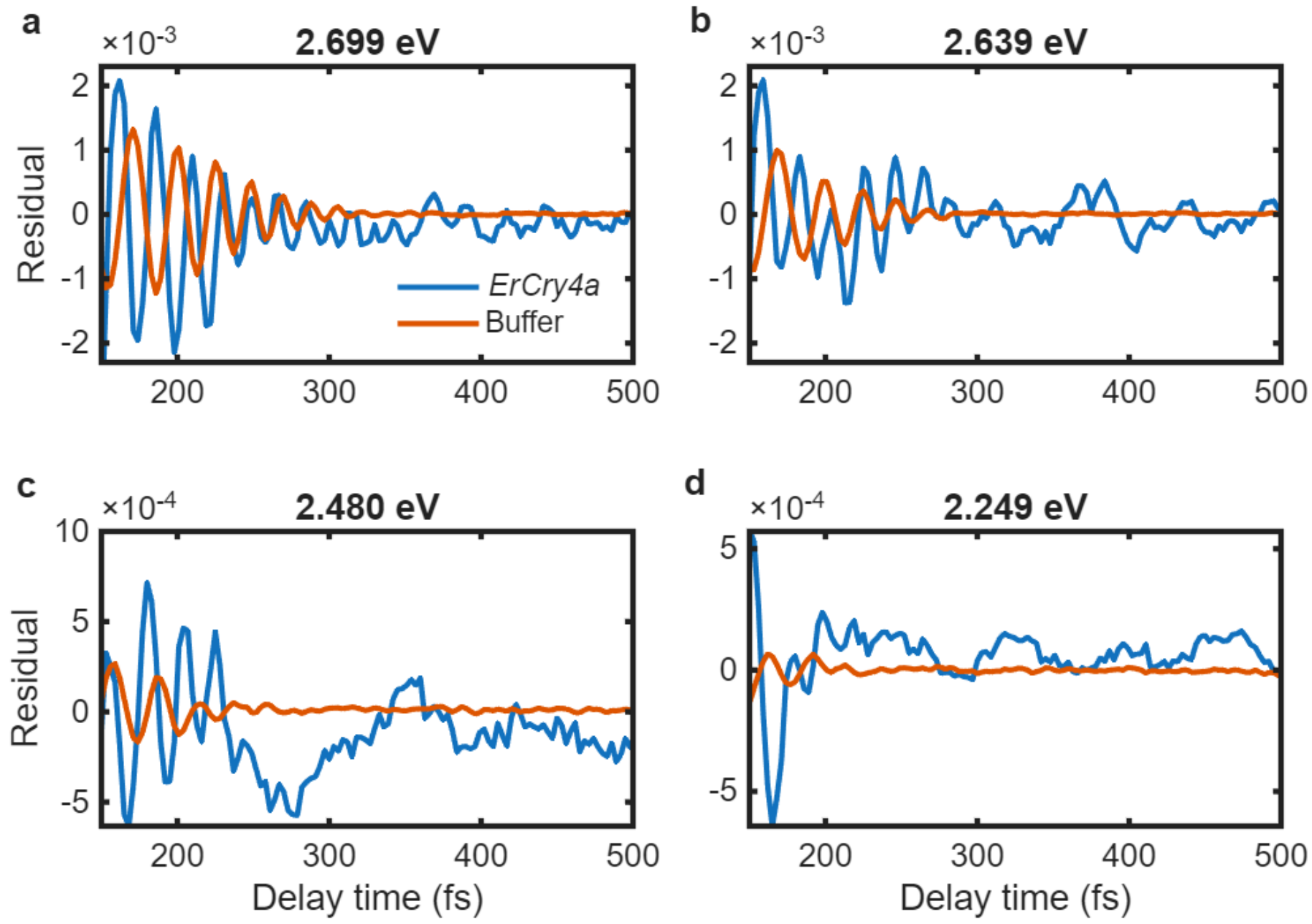

Figure S7. *Comparison of the XPM signal from the buffer measurement to the residuals from* Er*Cry4a measurements. a-d) Residuals obtained after subtracting the results from the global analysis from* Er*Cry4a data are plotted as a blue line at selected probe energies. The XPM signal from buffer, divided by 7, are shown in red.

Even after the buffer correction, the residuals of the *Er*Cry4a measurements during the first ~200 fs show a small amount of XPM signal since the total XPM signal is nearly an order of magnitude higher than the remaining pump-probe signal. This makes it difficult to fully correct all XPM contributions to the *Er*Cry4a data. The resulting residual map for *Er*Cry4a is shown in Fig. S5 and residuals at selected probe energies are displayed in Fig. S6. These data show damped oscillations which cannot be unambiguously assigned to either the XPM subtraction or other nonlinear coherent signals from the protein. To not affect the analysis of the vibrational coherences by these spurious signal contributions, residual maps (Fig. S5) in the delay range from 150 fs to 2200 fs are used for the Fourier transforms of the chirped probe data. Further, the data are filtered by multiplication with a normalized Gaussian function with 2.3 ps full width at half-maximum and zero-padded up to ~6 ps. A Fast Fourier transform results in FT maps that show the amplitude of the vibrational modes for each detection energy. This map can be used for quantitative analysis of amplitude and phase profile of the spectral mode for different vibrational modes. The use of chirped probe pulses results in an increased background in the FT spectra of the high frequency modes. Therefore, the chirped probe data are only used to analyze low-frequency modes. A comparison of the resulting spectrally integrated FT spectra for chirped probe and compressed probe is shown in Fig. S7.

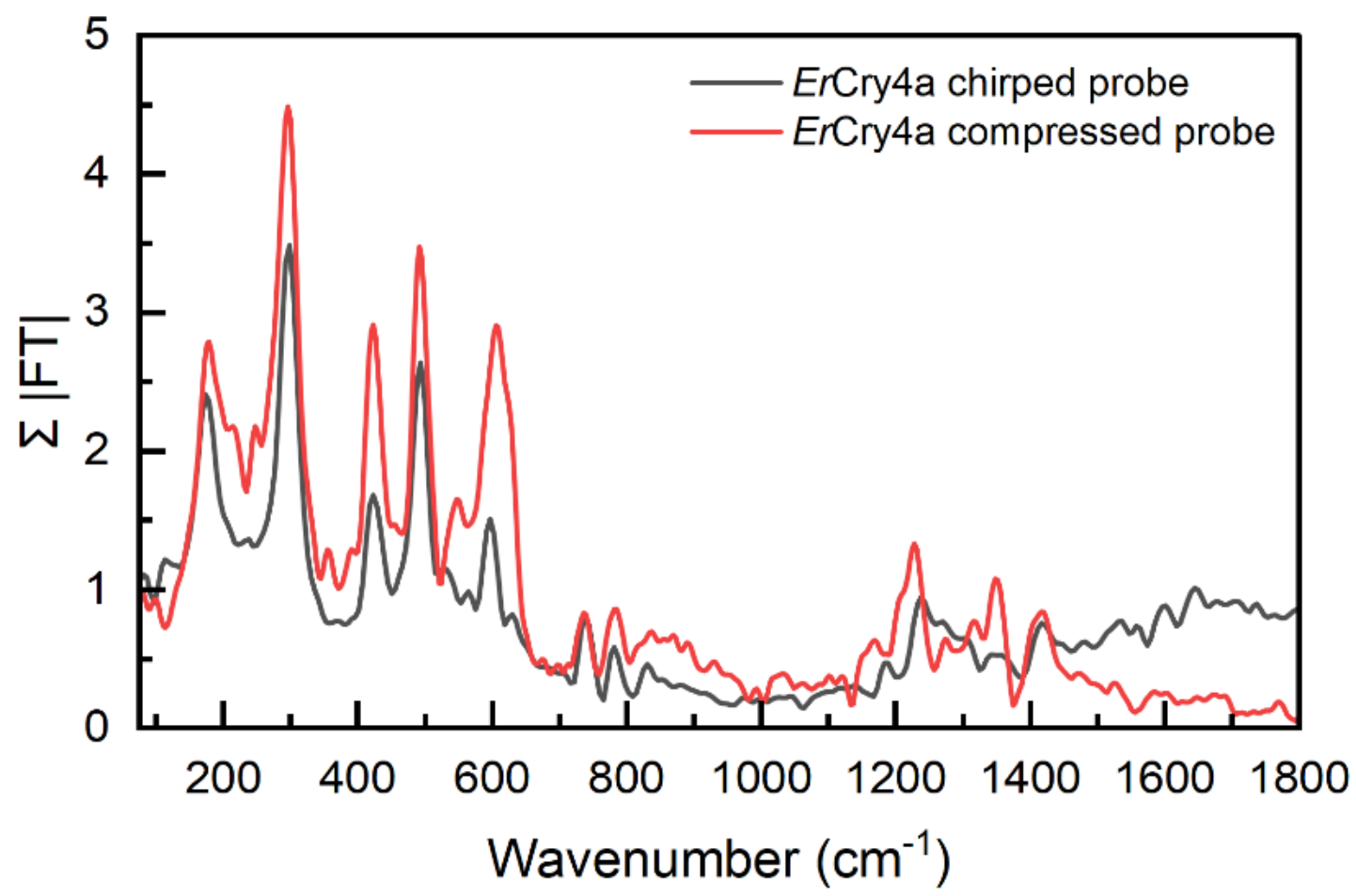

Figure S8. *Comparison of the Fourier transforms of coherent oscillations on the $\Delta T/T$ data, launched by pump excitation and measured by chirped probe (black) and compressed probe (red). The Fourier transform spectra are spectrally integrated along the probe energy axis. The Fourier analysis is performed on residuals from 200 fs, and the obtained oscillations are integrated over 2.14 – 2.75 eV*

3.4. Vibrational frequencies deduced from pump-probe spectroscopy

The frequencies of the modes observed in the chirped-probe experiments are listed in Table S1 and compared to published Raman modes of FAD.[8-11] Overall, a good agreement between vibrational energies can be seen with the literature reports.

Table S1: *Vibrational modes frequencies observed in this work is compared to literature reports.*

This work	Merk et al. (Ref [10])	Schelvis et al. (Ref [9])	Copeland et al. (Ref [8])	Weigel et al. (Ref [11])	Our work (FAD)[3]
1645	1625	1626	1634	1632	1632
1602	1579	1580	1584	1584	1585
1536	1550	1554	1552	1548	-
-	1505	1500	1505	1503	1509
1482	1480	1458	1483	1462	1484
1417	1422	1405	1426	1409	1412
-	1373	-	-	-	1388
1352	1332	1355	1340	1354	1349
1308	1305	1299	1310		1308
1270				1283	1283
-	1252	1247	1259	1256	1258
1238	1223	1231	1232	1230	1228
-	-	-	-		1201
1189	-	1181	1183	1182	
-	1160	1160	1169	1160	1154
-	-	-	1071	1066	1047
-	1008	-			1012
831	-	830		831	834
	-	-			818
782	790	789		788	782
738	732	743		741	739
	685	678			
630	609	629		610	607
597		600			
532		535		530	538
		?			518
494					489
423		431		428	424
		346			348
		315			
299				296	285
239				217	
179				184	185

3.5. Fourier maps

High-frequency modes analyzed based on TA maps recorded with compressed probe pulses:

The Fourier analysis of high-frequency vibrations observed using compressed probe data for FAD in water are shown below in Fig. S8 for qualitative comparison with the *Er*Cry4a data shown in Fig. 2 of main text. Coherent oscillations on the pump-probe maps persist for several 100 fs. These oscillations are induced by different high-frequency vibrational modes with frequencies between 1100 and 1700 cm^{-1}. The mode frequencies and their spectral profiles in the Fourier transform map are very similar to those observed in *Er*Cry4a proteins.

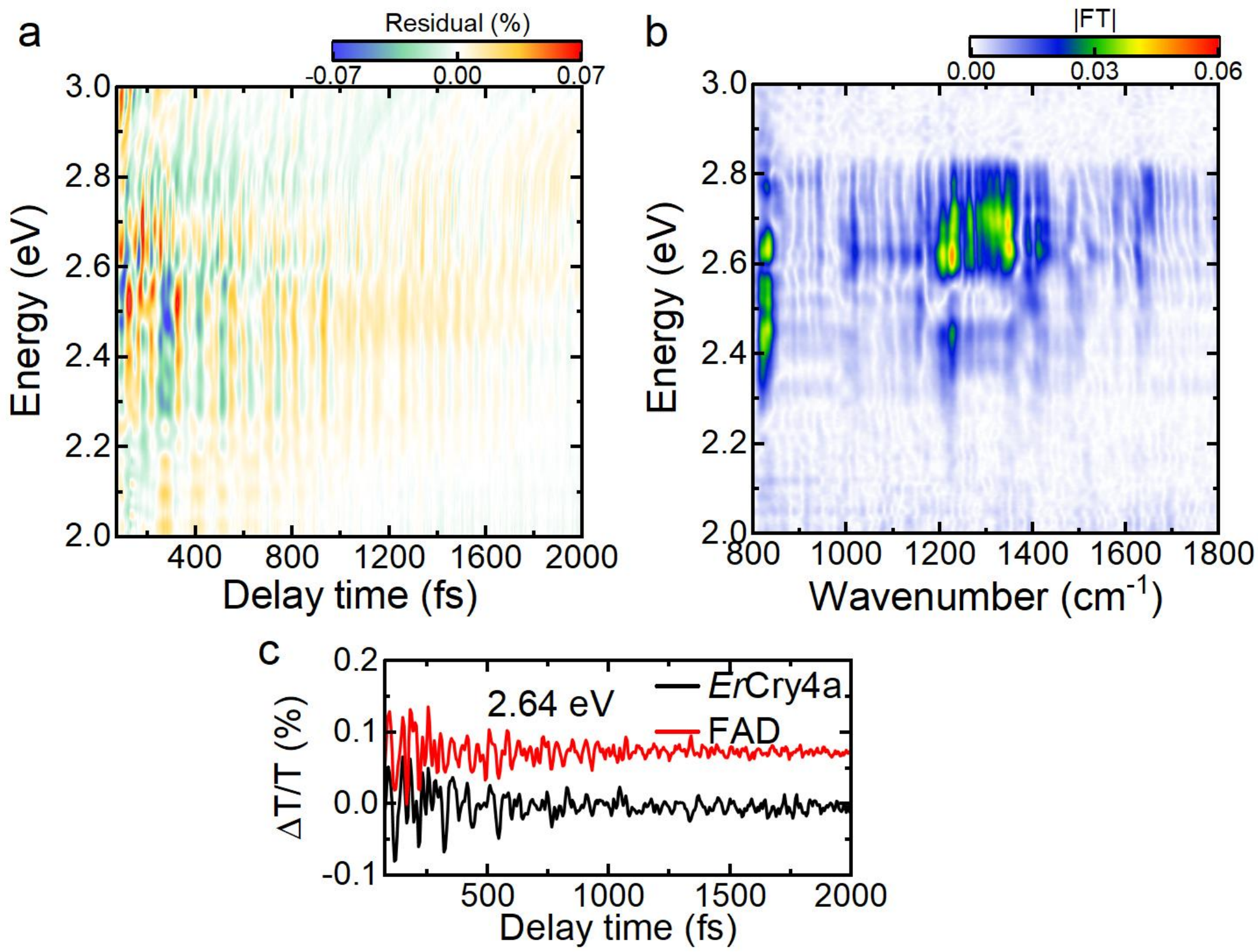

Figure S9*. Fourier analysis of high-frequency coherent oscillations induced by optical excitation in FAD. a) time-domain residuals map for free FAD extracted by subtracting exponential decays obtained by global fit. b) FT spectra of high-frequency vibrational modes in FAD deduced from a, c) Comparison of the residuals in FAD and in* Er*Cry4a at a probe energy of 2.64 eV. FAD residuals are vertically shifted.*

Low-frequency modes analyzed based on TA maps recorded with compressed probe pulses:

The Fourier analysis of low-frequency vibrations observed in the compressed probe data recorded for *Er*Cry4a and free FAD dissolved in water are shown below in Fig. S9. These data contain a finite sinusoidal chirp that is imprinted by the chirped mirrors on the probe pulses. This chirp affects the line shape of the FT spectra, inducing, e.g., minimum in the FT amplitude at certain probe energies. To avoid such complications, we chose chirped probe data for the qualitative evaluation of low-frequency modes in the main manuscript (Fig. 3).

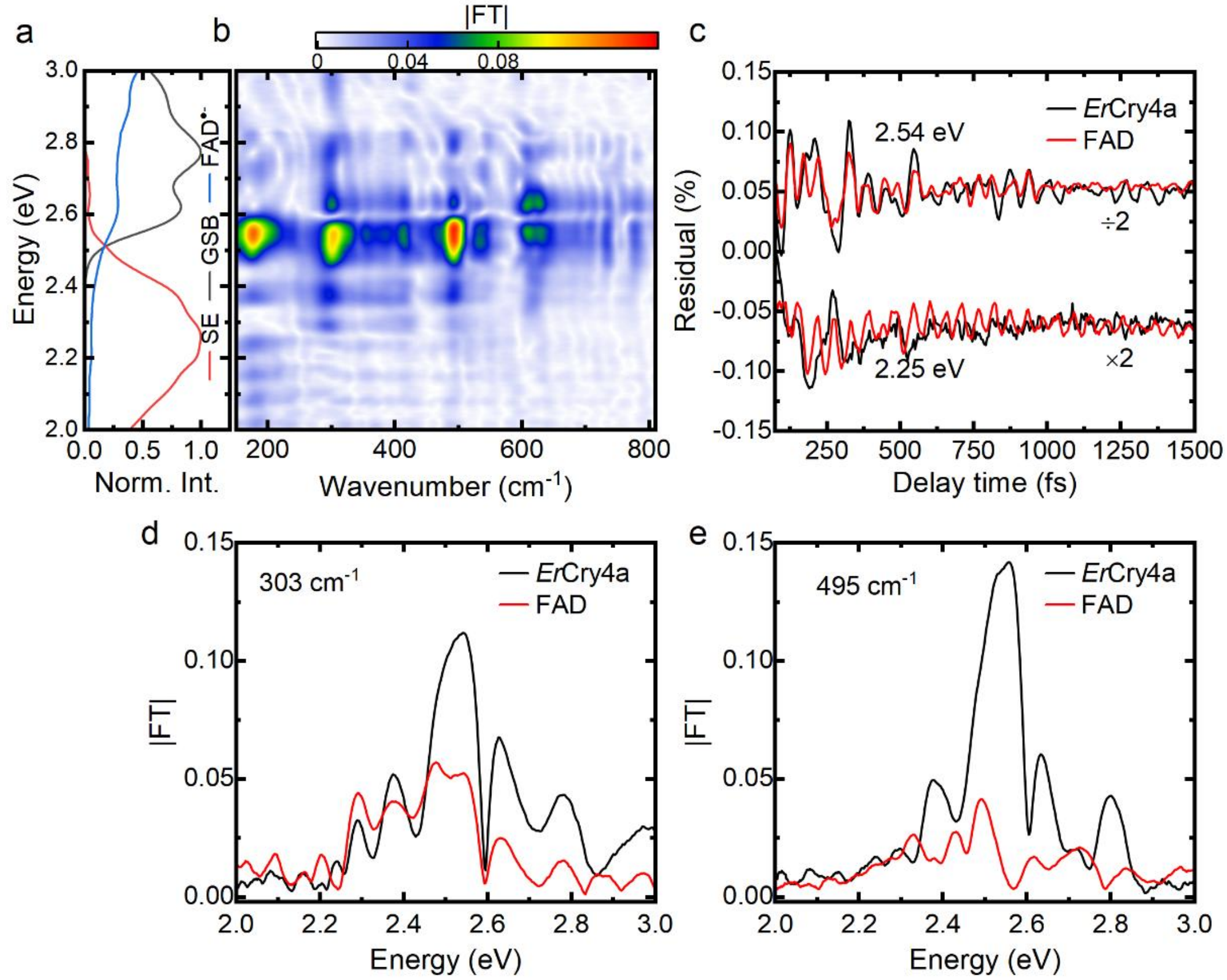

***Figure S10**. Fourier analysis of low-frequency coherent oscillations induced by optical excitation in Er*Cry4a*. a) Steady-state absorption (black) and emission (red) spectra of Er*Cry4a *and absorption spectrum of FAD•- (blue) obtained from literature.*[12] *b) Fourier transform map of Er*Cry4a *in the low-frequency region. c) Comparison of time-domain residuals at selected probe energies for Er*Cry4a *(black) and free FAD (red) extracted by subtracting exponential decays obtained by global fit. d, e) FT amplitude comparison for Er*Cry4a (black) *and free FAD (red) of selected low-frequency mode 303 cm*$^{-1}$ *(d), and 495 cm*$^{-1}$ *(e) plotted as a function of probe energy.*

Low-frequency modes analyzed based on TA maps recorded with chirped probe pulses:

The time-domain residual map and low-frequency Fourier map obtained from chirped probe data for FAD dissolved in water is shown below in Fig. S10 for comparison with *Er*Cry4a data in Fig. 3 of the main manuscript. The Fourier transformed map of FAD compared to *Er*Cry4a shows that the low-frequency modes below 2.4 eV are strongly suppressed showing the effect of electron transfer on these modes.

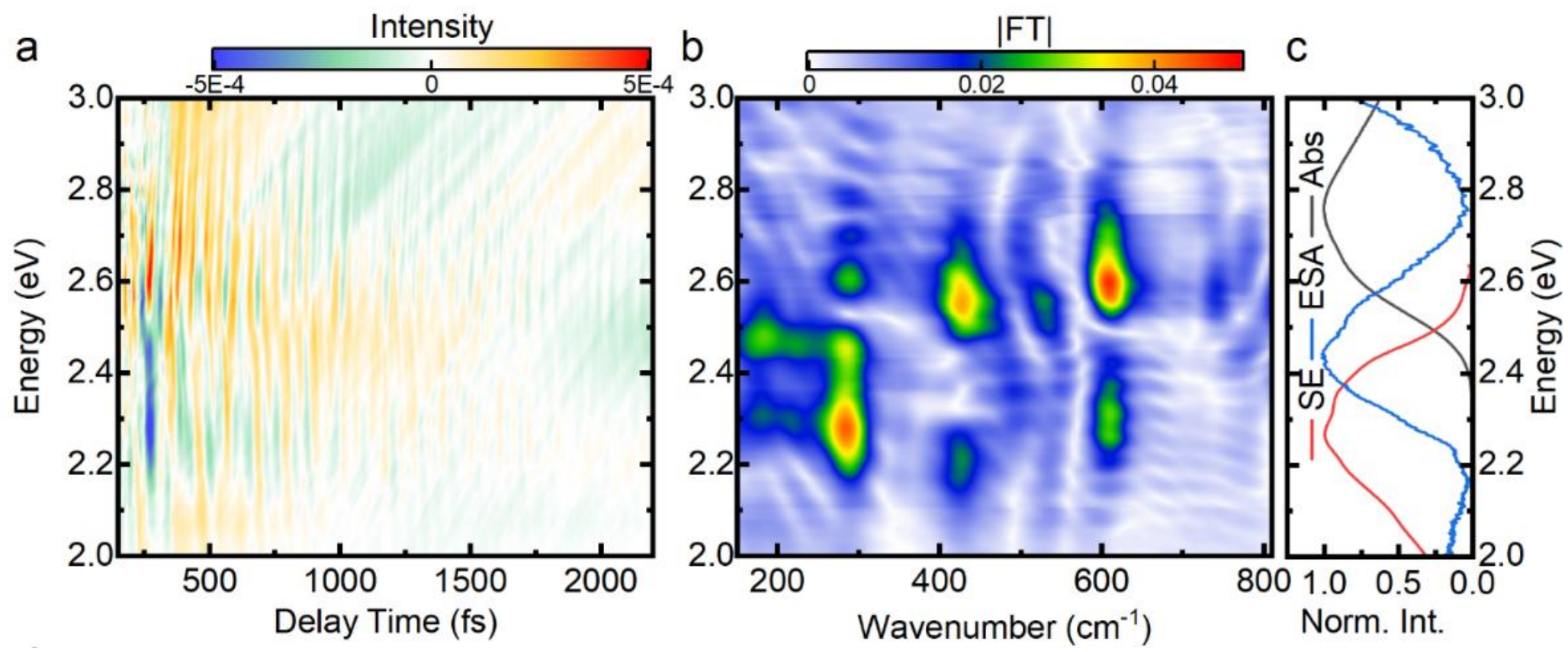

***Figure S11**. Fourier analysis of low-frequency coherent oscillations induced by optical excitation in FAD. a) Residuals extracted by subtracting exponential decays obtained by global fit. b) FT amplitude map of low-frequency mode plotted as a function of probe energy. c) Steady-state absorption (black) and emission (red) spectra and excited–state absorption spectrum (multiplied by -1) obtained by literature method for oxidized FAD.[11]*

*Mode profiles of low-frequency modes in Er*Cry4a:

The amplitude and phase profile of low-frequency modes for *Er*Cry4a (Fig. S11) measured using pump and probe pulses generated using a hollow-core fiber. The amplitude profile shows that the lower energy lobe below 2.4 eV is strongly suppressed due to the electron transfer from the nearby tryptophan unit.

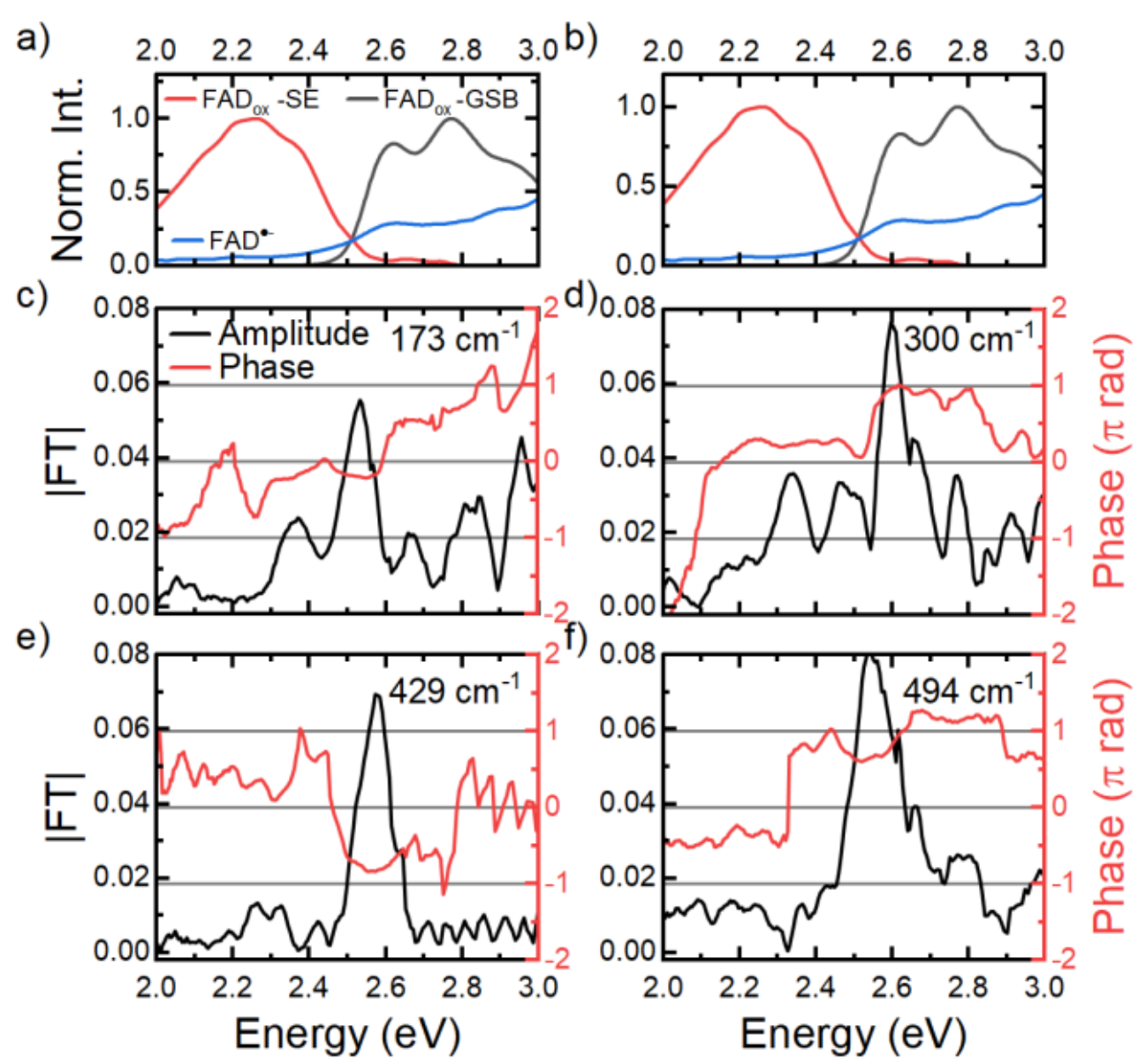

***Figure S12.** Mode profiles of low-frequency coherent oscillations induced by optical excitation in ErCry4a. a, b) Component spectra of different signal contributions in ΔT/T signal, GSB (black) same as steady-state absorption, SE same as emission (red) spectra and FAD$^{•-}$ obtained from literature.[12] c-f) amplitude (black), phase (red) of 173 cm^{-1} (c), 300 cm^{-1} (d), 429 cm^{-1} (e), and 494 cm^{-1} (f) modes.*

Mode profiles of low frequency modes of fully oxidized FAD in water:

Mode profiles of low frequency modes of FAD in water are shown in Fig. S12 in comparison with Fig S11. The amplitude profiles in case of FAD show a pronounced lower energy lobe around 2.2-2.3 eV since here the intramolecular ET from adenine in stacked conformation is slower than the decay of the vibrational oscillations on a picosecond timescale. Therefore, coherent excited state vibrational wavepacket motion associated with these modes in monitored via the SE transition centered around 2.2 eV.

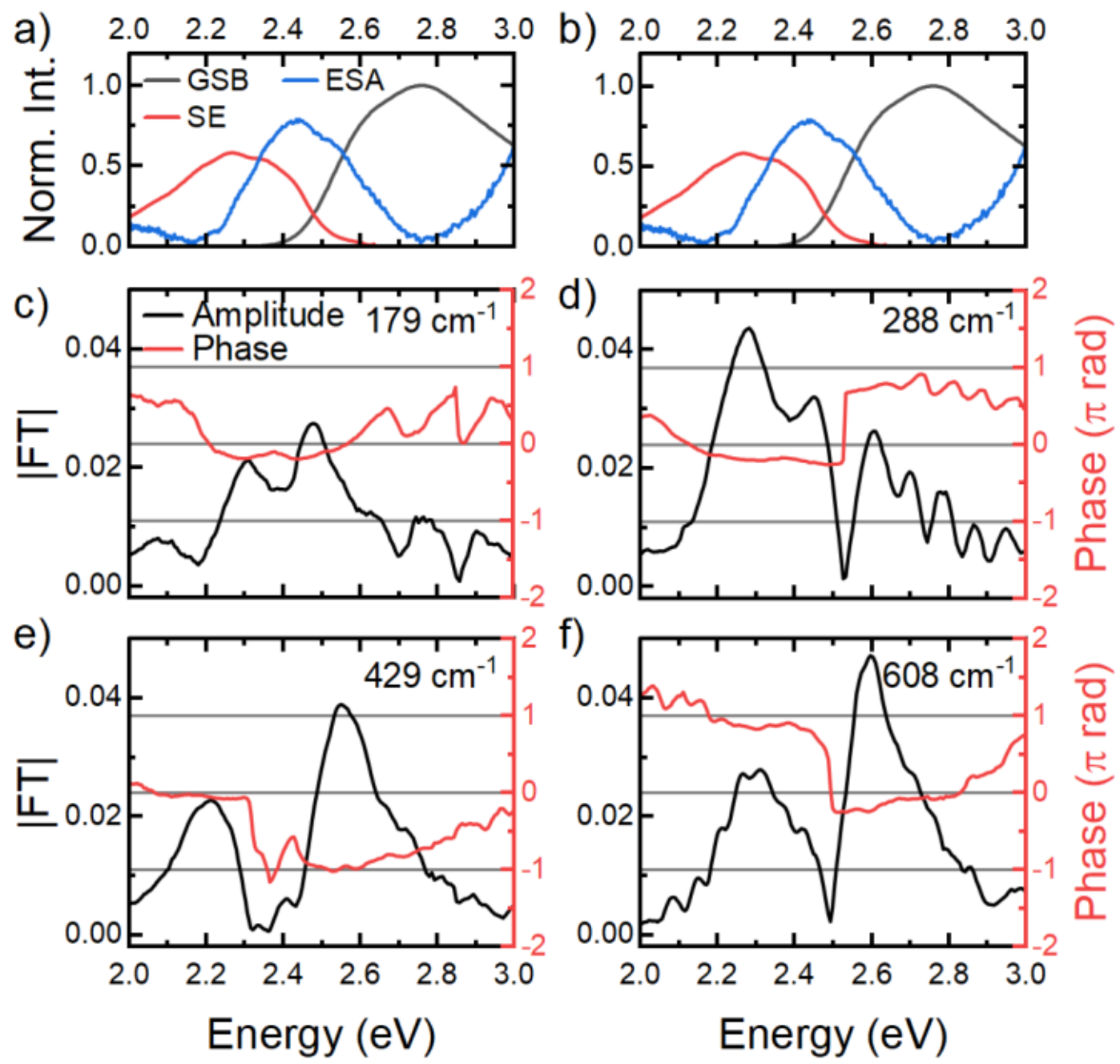

Figure S13. *Mode profiles of low-frequency coherent oscillations induced by optical excitation in fully oxidized FAD. a, b) Component spectra of different signal contributions in ΔT/T signal, GSB (black) same as steady-state absorption, SE same as emission (red) spectra and ESA (blue, multiplied by -1) obtained by literature method[11] c-f) amplitude (black), phase (red) of 179 cm^{-1} (c), 288 cm^{-1} (d), 429 cm^{-1} (e), and 608 cm^{-1} (f) modes.*

3.6. Theoretical calculations

All calculations of vibrational and UV/vis spectra employed a riboflavin (RF) or a riboflavin radical anion ($RF^{\bullet -}$) instead of the full FAD species to reduce computational costs. Density functional theory (DFT) with the CAM-B3LYP[13] functional was used with D3 correction and Becke-Johnson[14, 15] damping in conjunction with a def2-TZVP basis.[16] All calculations were done in implicit solvent (water) using a CPCM model.[17] For the optical spectra, time-dependent (TD-)DFT was employed[18, 19] as well as multireference DFT (DFT/MRCI) with the R2022 Hamiltonian.[20, 21] To account for model deficiencies, a scaling factor was applied to TD-DFT (0.805) and DFT/MRCI (0.91) predicted energies, matching the experimental S_1 peak. Spectral broadening was obtained using Lorentzians (IR, FWHM 25 cm^{-1}, Fig. S14) and Gaussians (UV/vis, FWHM 0.4 eV, Fig. S13).

Calculation of UV-visible spectra:

Both TD-DFT and DFT/MRCI reproduce the experimentally determined state energies of neutral and negative species (Fig 3a and Fig 4a, main text; see also ref. [22] of the main text for similar computational

results). Even though scaling has been applied to both flavin species based purely on matching the neutral species' S1 state to the theoretical results, the negative flavin species compares well to the experimental results: A shoulder is found near 2.6 eV and the maximum is located at 3.0 eV, as found by the measurements (see Figure S9a above and Figs. 3a and 4a of the main text).

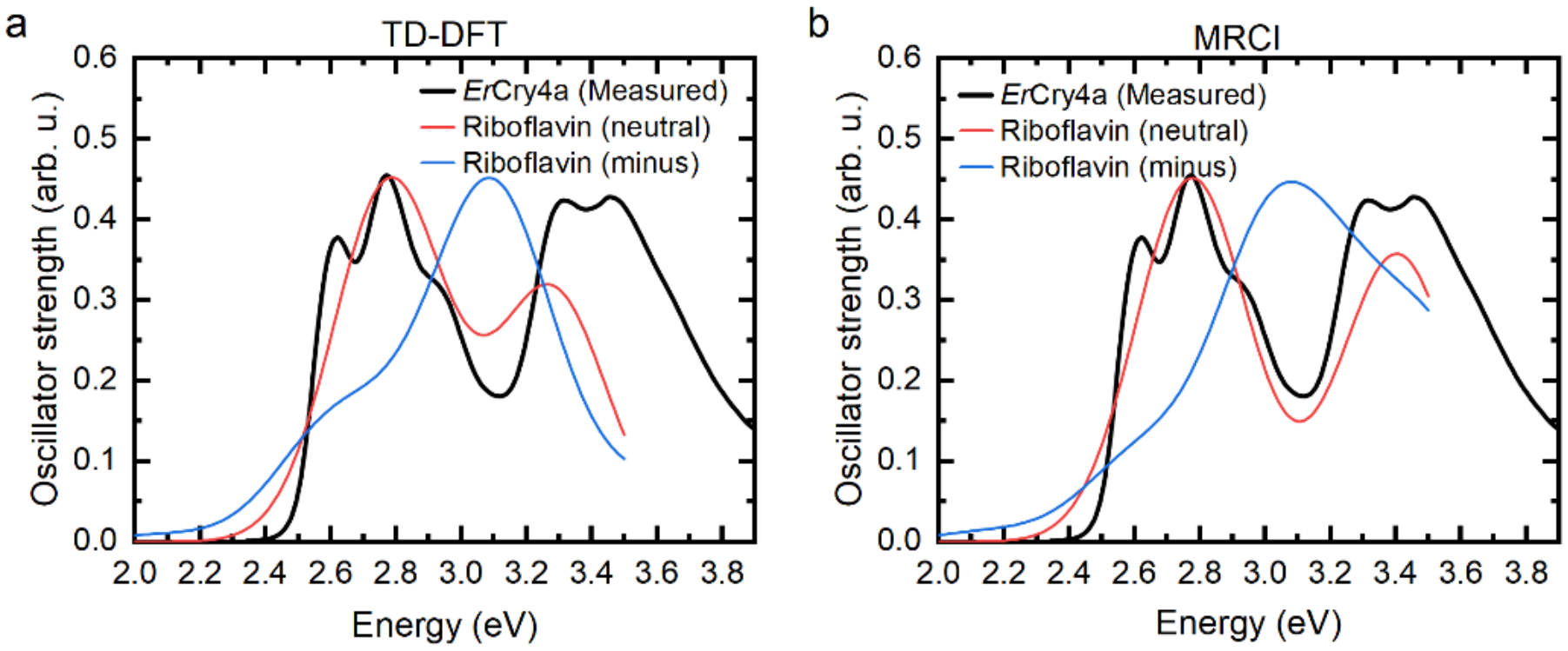

Figure S14*. Calculated electronic absorption spectrum of riboflavin. a) Comparison of the experimentally observed absorption spectrum of* ErCry4a *(black) and the absorption spectrum of neutral singlet riboflavin (red) and the negatively charged doublet riboflavin radical (blue) calculated using TD-DFT (a) and DFT/MRCI (b). Spectral broadening was obtained using a Gaussian function with a full width at half maximum of 0.4 eV.*

Calculations of IR spectra:

The computed infrared spectra (both for neutral and negative/radical flavin) agree quantitatively and qualitatively with those presented in ref [23] (data not shown), indicating that CAM-B3LYP is in good agreement with already reported computations and experiments. In Figure S14, we concentrate on the low-frequency region, which provides characteristic differences between the neutral and charged/radical form. The low-frequency calculated IR spectrum of the neutral form shows all major experimentally observed vibrational modes for FAD. Note that the spectrum in Figure S14 is not shifted; from ref. 23, a shift factor of 0.96875 can be deduced comparing our computations to experiment. As we present the unaltered IR results here, the computed peaks should therefore be located blue shifted from experimental peaks, as expected within the harmonic approximation.

Specifically, the modes at (experimental) 192 cm^{-1}, 429 cm^{-1}, 518 cm^{-1} and ~625 cm^{-1} can be assigned to deformation modes of the isoalloxazine ring. It is therefore no surprise that they are found in *Er*Cry4a as well, since the effect of the protein on this type of modes should be less than, e.g., vibrations involving hydrogen bridges. The latter is nicely seen via the modes near 600 cm-1, which have a higher intensity in free FAD than in *Er*Cry4a; those modes arise from ribityl-related hydrogen dihedral vibrations. As hydrogen bonding is only represented in an implicit solvent model here, the corresponding peaks are less reliable in energy and intensity than the others. Further, these hydrogen-bridging vibrations mix with a (calculated) energetically close isoalloxazine in-plane mode corresponding to the experimental shoulder near 625 cm^{-1}, reducing the energy of that peak in the calculations to 609 cm^{-1} (all other modes are computed to be at higher energies than experiment, as expected). Movies of the vibrational mode pattern are shown in Supplementary Information.

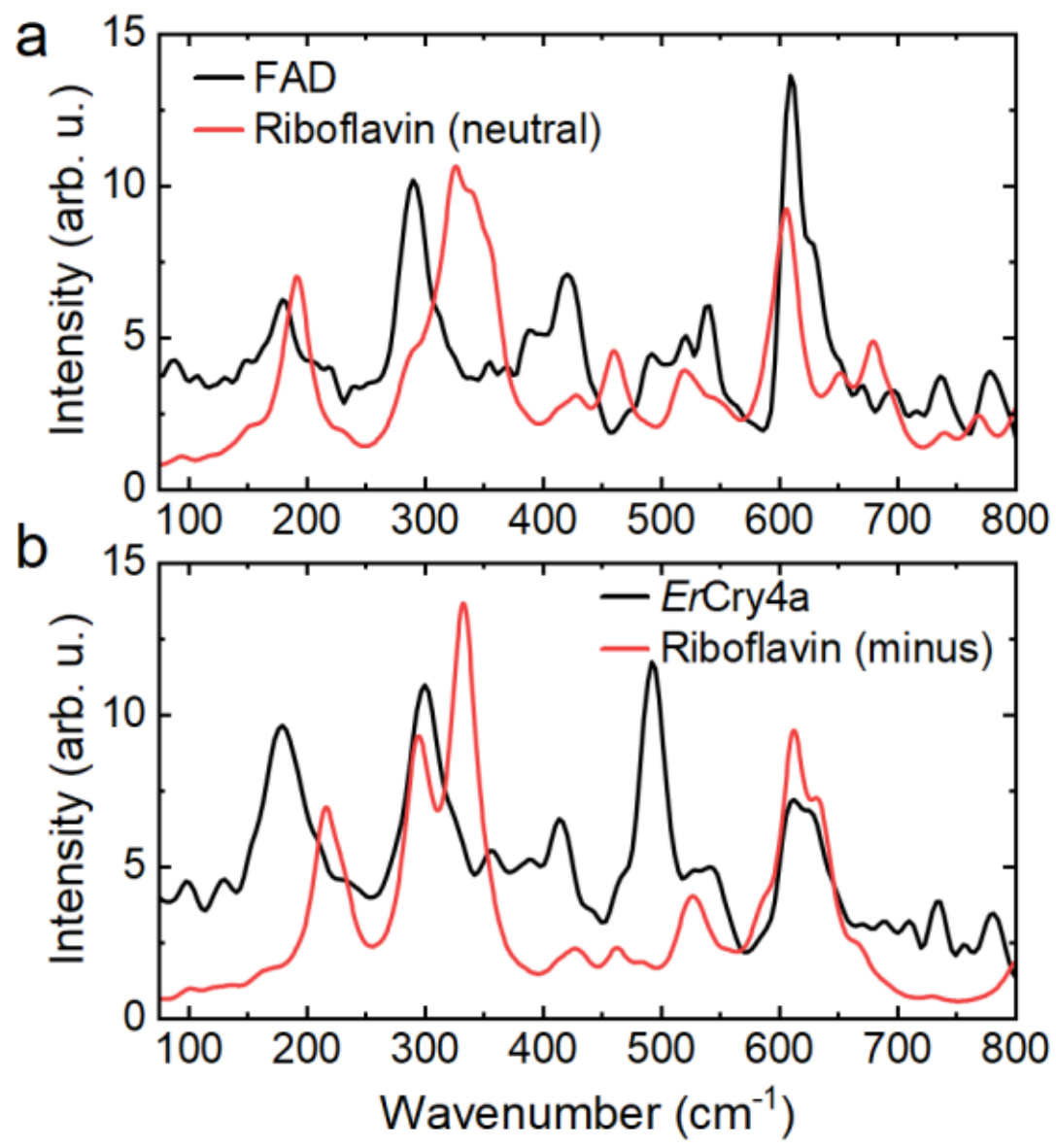

***Figure S15**. Comparison of the calculated IR and amplitude of the vibrational modes deduced from the FT analysis of Er*Cry4a*. a) Amplitude of the FT spectra, integrated over the probe energy for* Er*Cry4a (black). The data are taken from Fig. 2d of the main manuscript. The calculated IR spectrum of negatively charged riboflavin is shown as a red line. b) Amplitude of the FT spectra, integrated over the probe energy for FAD in water (black). The data are taken from Fig. 2d of the main manuscript. The calculated IR spectrum of charge neutral riboflavin is shown as a red line.*

4. References

(1) Xu, J. J.; Jarocha, L. E.; Zollitsch, T.; Konowalczyk, M.; Henbest, K. B.; Richert, S.; Golesworthy, M. J.; Schmidt, J.; Déjean, V.; Sowood, D. J. C.; Bassetto, M.; Luo, J. T.; Walton, J. R.; Fleming, J.; Wei, Y. J.; Pitcher, T. L.; Moise, G.; Herrmann, M.; Yin, H.; Wu, H. J.; Bartölke, R.; Käsehagen, S. J.; Horst, S.; Dautaj, G.; Murton, P. D. F.; Gehrckens, A. S.; Chelliah, Y.; Takahashi, J. S.; Koch, K. W.; Weber, S.; Solov'yov, I. A.; Xie, C.; Mackenzie, S. R.; Timmel, C. R.; Mouritsen, H.; Hore, P. J. Magnetic sensitivity of cryptochrome 4 from a migratory songbird. *Nature* **2021**, *594* (7864), 535-540.

(2) Timmer, D.; Frederiksen, A.; Lünemann, D. C.; Thomas, A. R.; Xu, J.; Bartölke, R.; Schmidt, J.; Kubař, T.; De Sio, A.; Solov'yov, I. A.; Mouritsen, H.; Lienau, C. Tracking the Electron Transfer Cascade in European Robin Cryptochrome 4 Mutants. *Journal of the American Chemical Society* **2023**, *145* (21), 11566-11578.

(3) Timmer, D.; Kumar, K.; Götze, J. P.; Saalfrank, P.; De Sio, A.; Lienau, C. Direct Observation of Franck–Condon Stimulated Emission and Sub-20 fs Relaxation in Photoexcited Flavins. *The Journal of Physical Chemistry Letters* **2026**, *17* (14), 4114-4124.

(4) Timmer, D.; Lünemann, D. C.; Riese, S.; De Sio, A.; Lienau, C. Full visible range two-dimensional electronic spectroscopy with high time resolution. *Optics Express* **2024**, *32* (1), 835-847.

(5) Sweetser, J. N.; Fittinghoff, D. N.; Trebino, R. Transient-grating frequency-resolved optical gating. *Optics Letters* **1997**, *22* (8), 519-521.

(6) Ekvall, K.; van der Meulen, P.; Dhollande, C.; Berg, L. E.; Pommeret, S.; Naskrecki, R.; Mialocq, J. C. Cross phase modulation artifact in liquid phase transient absorption spectroscopy. *Journal of Applied Physics* **2000**, *87* (5), 2340-2352.

(7) Rabe, M. Spectram: A MATLAB® and GNU Octave Toolbox for Transition Model Guided Deconvolution of Dynamic Spectroscopic Data. *J. Open Res. Softw.* **2020**, *8*, 13.

(8) Copeland, R. A.; Spiro, T. G. Ultraviolet resonance Raman spectroscopy of flavin mononucleotide and flavin-adenine dinucleotide. *The Journal of Physical Chemistry* **1986**, *90* (25), 6648-6654.

(9) Schelvis, J. P.; Pun, D.; Goyal, N.; Sokolova, O. Resonance Raman spectra of the neutral and anionic radical semiquinones of flavin adenine dinucleotide in glucose oxidase revisited. *Journal of Raman Spectroscopy* **2006**, *37* (8), 822-829.

(10) Merk, V.; Speiser, E.; Werncke, W.; Esser, N.; Kneipp, J. pH-Dependent Flavin Adenine Dinucleotide and Nicotinamide Adenine Dinucleotide Ultraviolet Resonance Raman (UVRR) Spectra at Intracellular Concentration. *Applied Spectroscopy* **2021**, *75* (8), 994-1002.

(11) Weigel, A.; Dobryakov, A.; Klaumünzer, B.; Sajadi, M.; Saalfrank, P.; Ernsting, N. P. Femtosecond Stimulated Raman Spectroscopy of Flavin after Optical Excitation. *Journal of Physical Chemistry B* **2011**, *115* (13), 3656-3680.

(12) Kao, Y. T.; Saxena, C.; He, T. F.; Guo, L. J.; Wang, L. J.; Sancar, A.; Zhong, D. P. Ultrafast dynamics of flavins in five redox states. *Journal of the American Chemical Society* **2008**, *130* (39), 13132-13139.

(13) Yanai, T.; Tew, D. P.; Handy, N. C. A new hybrid exchange-correlation functional using the Coulomb-attenuating method (CAM-B3LYP). *Chemical Physics Letters* **2004**, *393* (1-3), 51-57.

(14) Grimme, S.; Antony, J.; Ehrlich, S.; Krieg, H. A consistent and accurate ab initio parametrization of density functional dispersion correction (DFT-D) for the 94 elements H-Pu. *Journal of Chemical Physics* **2010**, *132* (15), 154104.

(15) Grimme, S.; Ehrlich, S.; Goerigk, L. Effect of the Damping Function in Dispersion Corrected Density Functional Theory. *Journal of Computational Chemistry* **2011**, *32* (7), 1456-1465.

(16) Weigend, F.; Ahlrichs, R. Balanced basis sets of split valence, triple zeta valence and quadruple zeta valence quality for H to Rn: Design and assessment of accuracy. *Physical Chemistry Chemical Physics* **2005**, *7* (18), 3297-3305.

(17) Barone, V.; Cossi, M. Quantum calculation of molecular energies and energy gradients in solution by a conductor solvent model. *Journal of Physical Chemistry A* **1998**, *102* (11), 1995-2001.

(18) Jamorski, C.; Casida, M. E.; Salahub, D. R. Dynamic polarizabilities and excitation spectra from a molecular implementation of time-dependent density-functional response theory: N-2 as a case study. *Journal of Chemical Physics* **1996**, *104* (13), 5134-5147.
(19) Casida, M. E.; Jamorski, C.; Casida, K. C.; Salahub, D. R. Molecular excitation energies to high-lying bound states from time-dependent density-functional response theory: Characterization and correction of the time-dependent local density approximation ionization threshold. *Journal of Chemical Physics* **1998**, *108* (11), 4439-4449.
(20) Grimme, S.; Waletzke, M. A combination of Kohn-Sham density functional theory and multi-reference configuration interaction methods. *Journal of Chemical Physics* **1999**, *111* (13), 5645-5655.
(21) Dombrowski, D. R.; Schulz, T.; Kleinschmidt, M.; Marian, C. M. R2022: A DFT/MRCI Ansatz with Improved Performance for Double Excitations. *Journal of Physical Chemistry A* **2023**, *127* (8), 2011-2025.
(22) Schwinn, K.; Ferré, N.; Huix-Rotllant, M. UV-visible absorption spectrum of FAD and its reduced forms embedded in a cryptochrome protein. *Physical Chemistry Chemical Physics* **2020**, *22* (22), 12447-12455.
(23) Le, D. P. N.; Hastings, G.; Gozem, S. Assigning flavin's difference-FTIR spectral bands in solution: frequency and intensity shifts in flavin's 1-electron and 2-electron reduced states. *Physical Chemistry Chemical Physics* **2025**, *27* (44), 24024-24039.
